# Identification of an Aging-Related Gene Signature in Predicting Prognosis and Indicating Tumor Immune Microenvironment in Breast Cancer

**DOI:** 10.3389/fonc.2021.796555

**Published:** 2021-12-16

**Authors:** Wenchang Lv, Chongru Zhao, Yufang Tan, Weijie Hu, Honghao Yu, Ning Zeng, Qi Zhang, Yiping Wu

**Affiliations:** Department of Plastic and Cosmetic Surgery, Tongji Hospital, Tongji Medical College, Huazhong University of Science and Technology, Wuhan, China

**Keywords:** breast cancer, aging-related genes, risk score, tumor immune microenvironment, prognosis

## Abstract

Breast cancer (BC) is the most commonly diagnosed malignancy accompanied by high invasion and metastasis features. Importantly, emerging studies have supported that aging is a key clue that participates in the immune state and development of BC. Nevertheless, there are no studies concerning the aging-related genes (AGs) in constructing the prognosis signature of BC. Here, to address this issue, we initially performed a systematic investigation of the associations between AGs and BC prognosis and accordingly constructed a prognosis risk model with 10 AGs including *PLAU, JUND, IL2RG, PCMT1, PTK2, HSPA8, NFKBIA, GCLC, PIK3CA*, and *DGAT1* by using the least absolute shrinkage and selection operator (LASSO) regression and Cox regression analysis. Meanwhile, our analysis further confirmed that the nomogram possessed a robust performance signature for predicting prognosis compared to clinical characteristics of BC patients, including age, clinical stage, and TNM staging. Moreover, the risk score was confirmed as an independent prognostic index of BC patients and was potentially correlated with immune scores, estimate score, immune cell infiltration level, tumor microenvironment, immunotherapy effect, and drug sensitivity. Furthermore, in the external clinical sample validation, AGs were expressed differentially in patients from different risk groups, and tumor-associated macrophage markers were elevated in high-risk BC tissues with more co-localization of AGs. In addition, the proliferation, transwell, and wound healing assays also confirmed the promoting effect of DGAT1 in BC cell proliferation and migration. Therefore, this well-established risk model could be used for predicting prognosis and immunotherapy in BC, thus providing a powerful instrument for combating BC.

## Introduction

Breast cancer (BC) in women has outstripped lung cancer as the most commonly diagnosed malignancy, with an estimated 2.3 million new cases (11.7%) around the world (1). The distant organ colonization caused by BC cells with high invasion and metastasis ability is the major threat to the management of advanced BC patients (2). BC encompasses disparate entities accompanied by various biomarkers and genetic signatures, thus leading to differences in prognosis among different BC subtypes (3). Therefore, reliable predictive biomarkers and the according predictive model are necessary for early precise diagnosis and individualized treatment for BC patients.

Aging is a complex multifactorial phenomenon manifested by the changes of the progressive loss of function or degeneration at every level of the human organism (4). There are many triggers in inducing aging, including telomere dysfunction, oxidase stress, DNA damage, and epigenetic changes (mouse models in modeling aging and cancer). Many investigations indicate that aging is supposed to be a key clue for many chronic diseases and a key clue in tumor evolution and mutation. In particular, aging is a comorbidity of BC that intensively implies the change of aging-associated transcriptome in promoting BC progression (5).

The aging process is highly influenced by genetic factors, which means multifarious alterations of aging-related genes (AGs) in the course of the disease (6). In some specific settings, the exonic variants in AGs are predictive events to phenotypic aging status. Emerging studies support that the alterations of AGs provide new meanings for pathogenesis, diagnosis, and treatment of cancer, diabetes, and other aging-related diseases (7). For example, Gnanavel et al. (8) identified a miRNA signature that was associated with aging-related senescence, in particular, signaling pathways associated with glioblastoma progression and proliferation. Oh et al. (9) also emphasized that the aged immune cells possessed the enhanced expression of five AGs, including *DUSP22, MAPK14, MAPKAPK3, STAT1*, and *VCP*, and promoted renal cell carcinoma invasion compared to young immune cells. Another interesting study also gives evidence to support that the aging-related signature was a confidential prognostic model with the capability of predicting the severity and immune cell infiltration of colorectal cancer patients (10).

**Figure 1:**
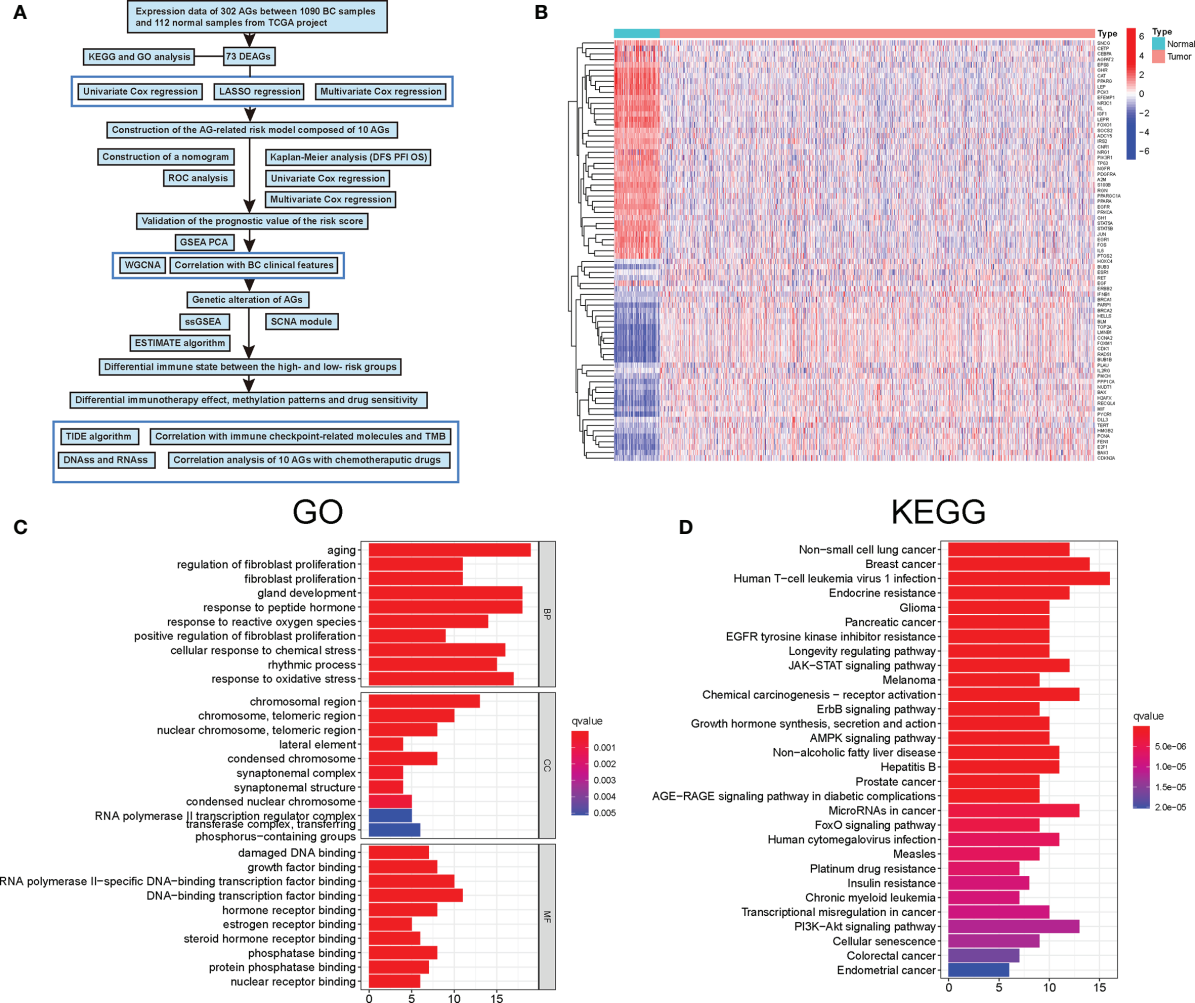
Characteristics and differences of aging-related genes (AGs) in breast cancer (BC). **(A)** Flowchart indicating the analysis procedure. **(B)** The expression of 73 differentially expressed aging-related genes (DEAGs) in BC tissues and normal tissues. **(C)** Gene Ontology (GO) enrichment analysis of 73 DEAGs. **(D)** Kyoto Encyclopedia of Genes and Genomes (KEGG) enrichment analysis of 73 DEAGs.

These studies indicated that the risk model based on AG alteration is of predictive performance and potential value in cancer prognosis. Although algorithms have been reported to predict prognostic or most age-related morbidity in BC, there were no systematic reports with regard to assessing the association of AGs and BC at present. In this study, we firstly obtained a total of 309 human AGs from the Human Ageing Genomic Resources. Then, the AG signature for prediction of BC clinical outcomes based on The Cancer Genome Atlas (TCGA) database was constructed using the least absolute shrinkage and selection operator (LASSO) regression and Cox regression analysis. We confirmed that the risk score was an independent prognostic index of BC patients in both the training and testing cohorts and was potentially correlated with immune scores, estimate score, immune cell infiltration level, tumor microenvironment, immunotherapy effect, and drug sensitivity (**Figure 1A**). More importantly, BC patients were divided successfully into the high- and low-risk groups based on the risk score. In addition to these two risk groups possessing different BC prognoses, they also exhibited disparate AG expression profiles and immune infiltrations. DGAT1 could also enhance the proliferation and migration of BC cells. Our research uncovered an underlying implication of AG-based signature, showing their potential as biomarkers for predicting clinical prognosis and immunotherapy for BC patients.

## Materials and Methods

### Acquisition of Sequencing Data and Clinical Data

Transcriptome sequencing data and corresponding clinical information of patients with BC were downloaded from TCGA database (https://portal.gdc.cancer.gov/). Subsequently, the “limma” R package in R software was applied to normalize the raw data of gene expression. A total of 1,202 samples (1,090 tumor samples and 112 normal samples) were selected for the following differential gene analysis. Then, the differentially expressed genes were used for Gene Ontology (GO) and Kyoto Encyclopedia of Genes and Genomes (KEGG) analyses. Meanwhile, clinical data including survival status, overall survival (OS), disease-free survival (DFS), progression-free interval (PFI), age, gender, stage, and TNM staging were also obtained from TCGA database, and samples lacking complete clinical data were excluded. All 1,090 tumor samples were set as the training set, and 544 BC samples were randomly selected as the internal testing set. A total of 20 patients who had undergone BC surgery were included in the external valid cohort, and the detailed clinical information of these enrolled BC patients was shown in **Table S1**.

### Construction and Validation of the Prognostic Aging-Related Gene Signature

A total of 309 human AGs were identified and obtained from the Human Ageing Genomic Resources and listed in **Table S2**. Firstly, based on the BC patient’s survival status and OS, the univariate Cox analyses were applied to identify prognosis-related AGs (11). Then, the LASSO regression was used to narrow down the AGs using the R package “glmnet.” Next, to avoid overfitting of models, the multivariate Cox regression analysis was performed to establish a signature for evaluating the relationships between the AGs and the BC patient’s OS (12). Finally, the risk score of each BC patient was calculated by the coefficient from multivariate Cox regression analysis, and then all patients were divided into high- and low-risk groups according to the median risk score (13). The formula of the risk score for the prediction of prognosis in BC patients was as follows: Risk score = coef gene (1) × exprgene (1) + coef gene (2) × exprgene (2) + **^…^** + coef gene (n) × exprgene (n). Then, BC patients in both the training set and testing set were separated into the high-risk and low-risk groups according to the median risk score cutoff value. In addition, Kaplan–Meier survival analysis was conducted to evaluate the differences of OS, DFS, and PFI in the two groups. Meanwhile, the log-rank test was used to calculate statistical significance, and P < 0.05 was considered significant. The clinical characteristics of the BC patients were combined with the prognostic AG signature to construct a nomogram through the “rms” R package. Then, the accuracy and discriminative power of the nomogram were evaluated by drawing a calibration.

### Establishment of Weighted Co-Expression Network

The weighed gene co-expression network analysis (WGCNA) algorithm was considered by establishing the scale-free co-expression network to explore the correlation between biologically meaningful gene modules and clinical characteristics (14). In this study, the R package “WGCNA” was run to calculate Pearson’s correlation matrices and adjacency function of all 309 human AGs. Then, to verify that the co-expression network presented a scale-free topology, parameter β (defined as the soft threshold of adjacency matrix) was used to penalize weak correlations and emphasize the strong correlations between AGs (15). After determining the appropriate β to be 6 based on ScaleFree plot, the adjacency matrix was transformed into a topological overlap matrix, and the new matrix followed the principle of scale-free topology (16). Finally, the co-expression gene modules (minModuleSize of 15) were identified to explore the functional modules associated with BC progression using the Dynamic Tree Cut algorithm.

### Gene Ontology, Kyoto Encyclopedia of Genes and Genomes, Gene Set Enrichment Analysis, Single-Sample Gene Set Enrichment Analysis, and Principal Component Analysis

Firstly, the GO and KEGG functional enrichment analyses based on differentially expressed AGs (DEAGs) were conducted through the R package “GOplot” and the R package “KEGGplot” (17). Secondly, gene set enrichment analysis (GSEA) was applied to explore the potential mechanisms and pathways between high- and low-risk patients using gsea-3.0.jar software (http://software.broadinstitute.org/gsea/index.jsp) (18). Then, the GO and KEGG analyses of the AG signature were conducted and visualized by the “enrichplot” and “clusterProfiler” R packages (19). The single-sample gene set enrichment analysis (ssGSEA) was applied to quantify the types of immune infiltrating cells between the two groups and identify their immune function through the “GSVA” R package (20). Moreover, we validated the classification results by performing the principal component analysis (PCA).

### Immune Cell Infiltration Analysis and Cancer-Immunity Cycle

The Cell-type Identification by Estimating Relative Subsets of RNA Transcripts (CIBERSORT) analysis with the “CIBERSORT” R package was applied to estimate the fractions of 22 immune cell types in all BC patients (21). Briefly, after filtering out genes with an average expression of 0, the proportion of multiple immune cells in normalized mRNAseq data was quantified using the leukocyte gene signature, termed LM22. LM22, consisting of 547 gene signatures, was able to distinguish 22 tumor-infiltrating immune cells, including different subtypes of B-cell, T-cell, natural killer cell, plasma cell, and myeloid cell types. Furthermore, the cancer-immunity cycle is a significant component of tumor immunotherapy research, during which the immune system suppresses cancer progression through seven specific steps. The genetic information of seven specific steps was downloaded from Tracking Tumor Immunophenotype (http://biocc.hrbmu.edu.cn/TIP/index.jsp) (13, 22).

### Tumor Microenvironment Analysis

According to the RNAssFile of “StemnessScores_RNAexp_20170127.2.tsv” and the DNAssFile = “StemnessScores_DNAmeth_20170210.tsv,” the tumor stem cell characteristics extracted from transcriptome and epigenetics of BC samples were used to evaluate stem cell-like features of tumors (23). The infiltration levels of immune cells and stromal cells in different samples were analyzed and quantized by immune score and stromal score. Meanwhile, tumor purity scores were estimated by the “ESTIMATE” R package (24). Moreover, the Pearson correlation was utilized to calculate the correlation between aging-related mRNA risk scores and infiltrating immune cells and tumor stemness.

### Immunotherapy/Chemotherapy Sensitivity Prediction

The tumor immune dysfunction and exclusion (TIDE) score was calculated online (http://tide.dfci.harvard.edu/) to assess the immune checkpoint inhibitor (ICI) response between high- and low-risk groups (25). The tumor mutation burden (TMB) was considered a marker for predicting immunotherapy response (26). On the basis of the prognostic AG signature, we explored TMB, somatic mutation, and copy number variations (CNVs) between high- and low-risk groups using the “maftools” R package. Moreover, the cBio Cancer Genomics Portal database (cBioPortal) was performed to assess mutations and CNVs in BC tissues. The different expressions of immune checkpoints between high- and low-risk groups could be used to predict the clinical response of corresponding inhibitors and provide new insights into clinical treatment. The NCI-60 database, consisting of 60 different cancer cell lines from nine different types of tumors, was performed to investigate drug sensitivity through the CallMiner software (https://discover.nci.nih.gov/cellminer) (27). Finally, the Pearson correlation analysis was utilized to detect the association between the prognostic AG expression and 263 drugs approved by the Food and Drug Administration (FDA).

### Cell Culture, RNA Interference, and Quantitative Real-Time PCR

The human mammary carcinoma cell lines MCF-7 and MDA-MB-231 were acquired from American Type Culture Collection (Manassas, VA, USA). Dulbecco’s modified Eagle’s medium (DMEM; Gibco, Carlsbad, CA, USA) supplemented with 10% fetal bovine serum (FBS; Gibco, Carlsbad, CA, USA) was utilized for the culture of MDA-MB-231 and MCF-7 cells at 37°C in 5% CO_2_ atmosphere.

The small interference RNAs (siRNAs) targeting *DGAT1* and negative control were designed and synthesized by Ribo Biotech (Guangzhou, China). The siRNAs were transfected into MDA-MB-231 and MCF-7 cells using Lipofectamine 3000 Transfection Reagent (Invitrogen, CA, USA) according to the manufacturer’s protocols. Then, RT-PCR was used to evaluate transfection efficiency after 24 h.

Total RNAs of cultured BC cells and tissue specimens were extracted by TRIzol (Takara, Japan), and cDNA synthesis with the 1st Strand cDNA Synthesis Kit (Yeasen, Shanghai, China) was performed based on the manufacturer’s protocols. The qRT-PCR was carried out with QuantStudio1 (ABI Q1, USA) using SYBR GreenTM Master Mix (Yeasen, Shanghai, China). All primer sequences used for qRT-PCR can be obtained in **Table S3**.

### Cell Proliferation, Transwell, and Wound Healing Assay

The proliferation abilities of MDA-MB-231 and MCF-7 cells were assessed using a Cell Counting Kit-8 (CCK-8) assay (Yeasen, Shanghai, China) according to the manufacturer’s instructions. Briefly, MDA-MB-231 and MCF-7 cells were seeded in a 96-well plate and incubated to 40% confluence, respectively. After transfection, 10-μl CCK-8 reagent was directly added to each well of a 96-well plate at the specified time (0, 12, 24, 36, 48, 60, and 72 h) and then incubated at 37°C for 1.5 h. Finally, optional density (OD) was measured by a microplate reader (BioTek Instruments, VT, USA) at 450 nm.

Transwell migration assays were performed using the 24-well transwell migration chambers (8-μm pore size; Corning, NY, USA). MDA-MB-231 and MCF-7 cells were resuspended in 200-μl serum-free DMEM and seeded into the inner chambers. Here, 500-μl DMEM medium with 20%FBS was added to the bottom chambers as an attractant. After 24 h of incubation, the membranes of the chamber were fixed with 4% paraformaldehyde for 30 min and the upper side of the chamber was wiped with cotton swabs to remove the unmigrated cells. Then, 0.1% crystal violet was used to stain the migrated cells on the lower side of the membrane for 10 min at 37°C. The number of migrated cells was counted by the ImageJ software.

Wound healing assay was performed to detect the migration capabilities of cells following siRNA transfection. After transfection, MDA-MB-231 and MCF-7 cells were plated on the six-well plate and grown to 90% confluence until forming a cell monolayer. A straight scratch was made on the single-cell layer in each well by a 200-μl micropipette tip. Next, phosphate-buffered saline (PBS) was applied to wash away the detached cells and debris, and then the cells were cultured in a serum-free DMEM medium. The horizontal distances of the migrating cells were photographed by microscopy for 24 h and were measured by ImageJ software.

### Immunohistochemistry and Immunofluorescence

For immunohistochemistry (IHC), human mammary tissues were deparaffinized in xylene and rehydrated in different concentrations of ethanol solutions and were heated in citrate buffer using the pressure cooker for antigen retrieval. Next, the sections were immune-stained with primary antibodies against human NFKBIA, PLAU, and DGAT1 (1:100; Proteintech, China) overnight at 4°C followed by horseradish peroxidase (HRP)-conjugated secondary antibodies. Peroxidase activity was visualized using a DAB Peroxidase Substrate Kit (Maxin, China), and the sections were counterstained with hematoxylin. The SOPTOP CX40 microscope (China) was applied to capture digital images of the sections.

For immunofluorescence (IF), the procedures of managing human mammary tissues before staining were the same as IHC. The mammary sections were incubated overnight at 4°C with a cocktail of primary antibodies against human NFKBIA and PLAU (1:100; Proteintech, China), followed by incubation with a cocktail of secondary antibodies (Life Technologies, CA, USA) for 1 h at room temperature on the next day. The sections were then incubated with human anti-CD206 (1:100; Proteintech, China) and nuclear 4,6-diamidino-2-phenylindole (DAPI; Vector Laboratories, Burlingame) for counterstaining. Digital images were captured by using the Olympus fluorescence microscope (Japan).

### Statistical Analyses

The Cox regression and LASSO regression analyses were conducted to identify the aging-related mRNAs and the independent prognostic factors in BC patients. The Kaplan–Meier curves and two-sided log-rank test were utilized to compare the OS between subgroups in BC patients. Moreover, we calculated receiver operating characteristic (ROC) curves, time-dependent ROC curves, and area under the curves (AUC) through the “pROC” and the “timeROC” packages, respectively (28). All statistical analyses were conducted using R version 3.6.1 with corresponding R packages. Experiments in this study were performed in triplicate with the statistical results presented as means ± standard deviation (SD) using GraphPad Prism Software (version 8.0.1; La Jolla, CA, USA). Student’s t-test was applied to compare the differences between the two groups. For all the data, ^∗^P < 0.05, ^∗∗^P < 0.01, and ^∗∗∗^P < 0.001 were considered statistically significant between the high- and low-risk groups.

## Results

### Identification of Differentially Expressed Aging-Related Genes in Normal and Breast Cancer Samples and Functional Analyses

A total of 309 human AGs were firstly obtained from the Human Ageing Genomic Resources. Then, among them, nearly 73 DEAGs were further identified between 1,066 BC and 112 normal samples, including 40 upregulated and 33 downregulated DEAGs [false discovery rate (FDR) <0.05 and |logFC| >1] (**Figure 1B**). Furthermore, the KEGG and GO pathway analyses were performed to validate the potential function of those DEAGs. The results of the GO analysis showed that those DEAGs were significantly associated with the biological process of aging and development of cellular senescence and cancer, including the response to peptide hormone, gland development, and response to oxidative stress (**Figure 1C**). Of interest, in the KEGG analysis, those DEAGs had a significant correlation with multiple cancers, in particular with BC (**Figure 1D**). Meanwhile, the KEGG analysis also revealed that those DEAGs were strongly related to human T-cell leukemia virus 1 infection. These results uncovered the vital role of AGs in the progression of cancer.

### Construction and Validation of the Aging-Related Gene-Related Risk Model With Prognostic Value in Breast Cancer

**Figure 2:**
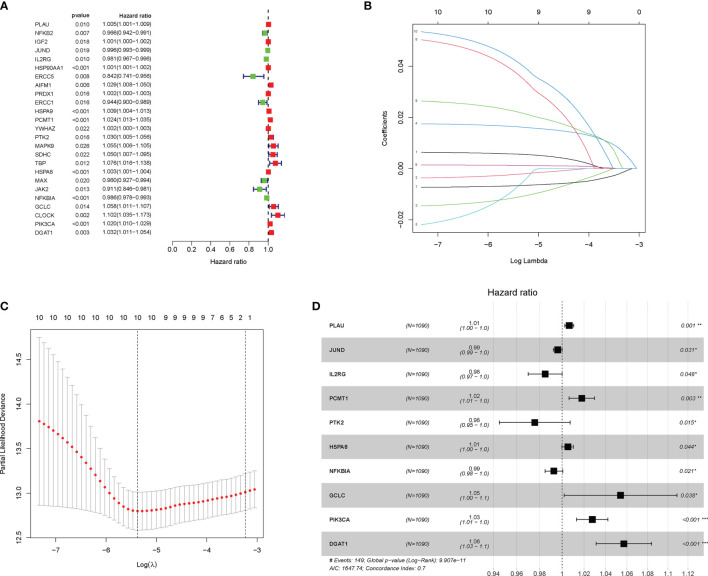
Construction and validation of the aging-related gene (AG)-related risk model with prognostic value in breast cancer (BC). **(A)** The prognostic genes associated with overall survival (OS) were identified by the univariate Cox analyses. **(B, C)** In the least absolute shrinkage and selection operator (LASSO) regression analysis of The Cancer Genome Atlas (TCGA), the value of the super parameter λ was verified by 10-fold cross-validation. **(D)** Forest plot showing the hazard ratio (HR) for the association between prognostic ability of the 10 AGs and OS. *P < 0.05; **P < 0.01; ***P < 0.001.

Then, the univariate Cox regression analysis was executed to select 25 AGs, which were significantly correlated with BC prognosis (P < 0.05) (**Figure 2A**). Then, the LASSO regression analysis and stepwise multivariate Cox regression analysis were performed to construct a prognostic gene model. The model with a λ was identified as the final model, which was composed of 10 AGs, including *PLAU, JUND, IL2RG, PCMT1, PTK2, HSPA8, NFKBIA, GCLC, PIK3CA*, and *DGAT1* (**Figures 2B**–**D**). The risk score was calculated by using the coefficients of those 10 AGs. The model formula was as follows: The risk score = *PLAU* × (0.0065) + *JUND* × (-0.0038) + *IL2RG* × (-0.0154) + *PCMT1* × (0.0177) + *PTK2* × (-0.0237) + *HSPA8* × (0.0014) + *NFKBIA* × (-0.0071) + *GCLC* × (0.0535) + *PIK3CA* × (0.0273) + *DGAT1* × (0.0558). Meanwhile, the Kaplan–Meier analysis further showed that among those 10 AGs, *PLAU, PCMT1, PIK3CA, PTK2, HSPA8*, *GCLC*, and *DGAT1* were correlated with poor OS of BC patients, while the high expression of *JUND, IL2RG*, and *NFKBIA* supported a longer survival time of BC patients (**Figure S1**).

**Figure 3:**
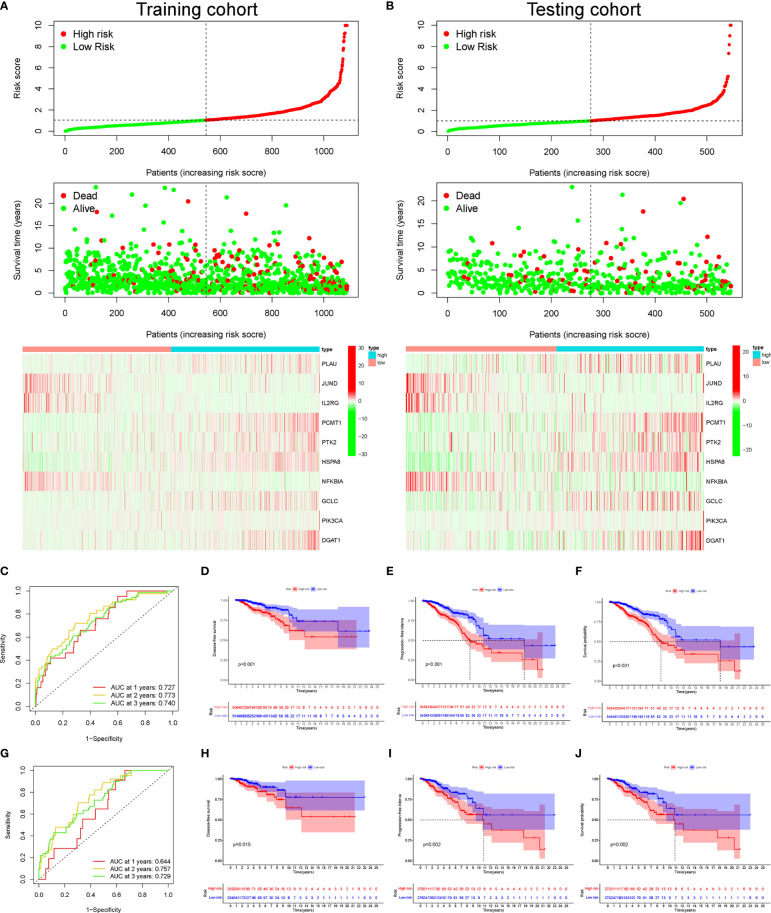
The distribution of risk score, survival status, and heatmap of mRNA expression panel. The time-dependent receiver operating characteristic (ROC) curve analysis and Kaplan–Meier curve of the 10 aging-related gene (AG) signature in The Cancer Genome Atlas (TCGA) datasets. The distribution of risk score, survival status, and the 10 AG expression panel of breast cancer (BC) patients with different risk scores in the training cohort **(A)** and testing cohort **(B)**. The green and red dots represented survival and death, respectively. Time-dependent ROC curve analysis in the training cohort **(C)** and testing cohort **(G)**. The Kaplan–Meier survival analysis showing the disease-free survival (DFS), progression-free interval (PFI), and the overall survival (OS) of BC patients between high- and low-risk groups in the training cohort **(D–F)** and testing cohort **(H–J)**.

Subsequently, according to the median threshold of the risk score, a total of 1,090 BC patients in the training set were divided into high-risk (545 patients) and low-risk groups (545 patients) (**Figure 3A**). As shown in **Figure 3A**, the death probability of BC patients augmented as the risk score increased. Besides, the heatmap visualized the expression pattern of those 10 AGs between the two risk subgroups. Notably, the ROC curve showed the optimal predictability of BC prognosis by the risk score, with the AUC of 0.727, 0.773, and 0.740 in 1, 2, and 3 years, respectively (**Figure 3C**). Meanwhile, the Kaplan–Meier curve uncovered that BC patients in the high-risk group had shorter DFS (**Figure 3D**), PFI (**Figure 3E**), and OS (**Figure 3F**) than those in patients in the low-risk group. Similarly in **Figure 3B**, based on the risk score, 544 BC patients in the testing cohort were also divided into high-risk (277 patients) and low-risk groups (267 patients). The ROC curve showed the similar predictability of BC prognosis by the risk score in the training set, with the ﻿AUC of 0.644, 0.757, and 0.729 in 1-, 2-, and 3-year, respectively (**Figure 3G**). Notably, the DFS (**Figure 3H**), PFI (**Figure 3I**), and OS (**Figure 3J**) of BC patients were lower in the high-risk group than those in the low-risk group in the internal testing cohort.

Furthermore, the external validation in other datasets was performed to validate the reliability of the AG signature in predicting prognosis through ROC curve analysis. The AUC value of our risk model signature for 3-year OS was higher when compared with other published prognostic signatures in BC (**Figure S2A**). The results showed that our risk model possessed the optimal predictive ability in the long-term survival rate of BC patients. In addition, the C-index of our risk model was also higher in comparison to others, illuminating the fairly stable characteristics of our risk model in predicting the prognosis of BC (**Figure S2B**). The Kaplan–Meier analysis indicated that the OS was significantly different between the high- and low-risk groups of BC patients according to our risk model and other risk signatures (**Figures S2C, D**). All the above results proved the better prognostic value of AG signature.

### Development of a Nomogram of Breast Cancer Patients

**Figure 4:**
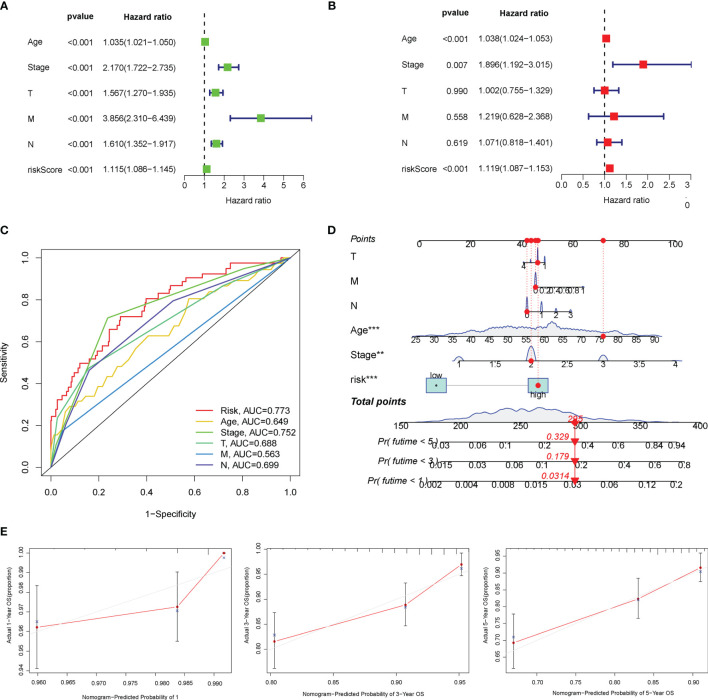
Overall survival (OS)-related factors were screened through the univariate and multivariate Cox analyses, and the prognostic accuracy of risk score, age, stage, and TNM staging was compared. The univariate **(A)** and multivariate **(B)** Cox regression analyses of the risk score and other clinical feature prognostic values. **(C)** The prognostic accuracy of risk score, age, stage, and TNM staging was compared in 2 years using the time-dependent receiver operating characteristic (ROC) curve. **(D)** Construction of a nomogram containing the risk score, age, stage, and TNM staging to predict 1-, 3-, and 5-year OS of breast cancer (BC). **(E)** The calibration curve for the prediction and observed 1-, 3-, and 5-year OS.

Furthermore, the univariate and multivariate Cox regression analyses were performed to explore whether the prognostic efficiency of the risk score was independent of other clinicopathological parameters, including age, clinical stage, and TNM staging. The univariate Cox regression analysis revealed that the risk score served as an independent risk factor of BC prognosis [hazard ratio (HR) = 1.115, 95% CI: 1.086–1.145, P < 0.001] (**Figure 4A**). After adjustment for other confounding factors, the risk score, age, and clinical stage were independently associated with poor OS of BC patients (P < 0.01) (**Figure 4B**). Additionally, compared with other clinicopathological factors, the AUC of the risk score for 2-year OS shown by ROC curve analysis reached 0.773, which was superior to those of other independent clinicopathological variables (**Figure 4C**). Next, we enrolled all independent factors to construct a nomogram for predicting the 1-, 3-, and 5-year OS of BC (**Figure 4D**). The nomogram was verified to be reliable and accurate by the calibration curve analysis, which demonstrated that the predictive probability of 1-, 3-, and 5-year OS was ideally consistent with actual observation (**Figure 4E**).

### Weighted Gene Co-Expression Network Analysis and Identification of Significant Modules With Breast Cancer Prognosis

**Figure 5:**
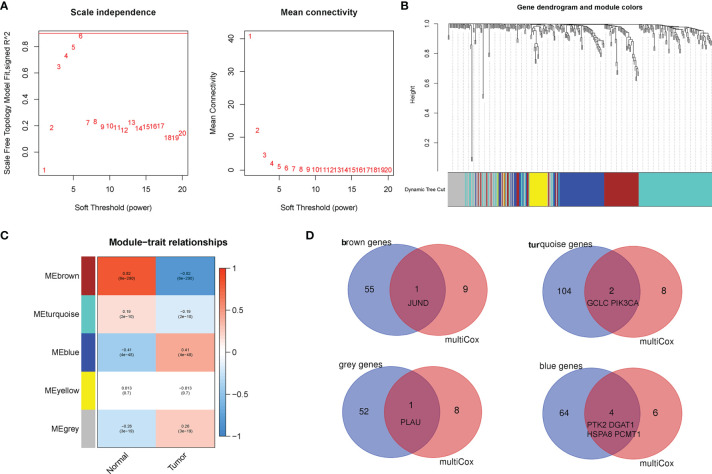
Establishment of weighted co-expression network and identification of significant modules with breast cancer (BC) prognosis. **(A)** The scale-free fit index for various soft-thresholding powers (β). The power of β = 6 was set as the soft-thresholding parameter to meet the scale-free topology. **(B)** Dendrogram of all aging-related genes (AGs) clustered based on a dissimilarity measure. **(C)** Identifying co-expression modules associated with the BC progression. The red module represented a positive correlation with clinical features, while the blue module represented a negative correlation with clinical features. **(D)** The Venn diagram revealed that eight AGs among the aging-related risk model were identified to be related to BC.

Subsequently, the WGCNA was constructed to identify the correlations among AGs through undirected networks and stepwise to obtain modules of highly correlated genes with different clinical features. In our study, the power of β = 6 was set as the soft-thresholding parameter to meet the scale-free topology (R^2^ > 0.9) (**Figure 5A**). Then, the hierarchical clustering tree and the recognition co-expression network were established according to the cutoff value = 0.25 (**Figure 5B**). Finally, a total of five gene modules of AGs were identified, and the minimum size of the gene group was 15. Furthermore, the eigengene of each module was calculated to correlate the modules with the corresponding clinical characteristics (tumor or normal). The red module represented a positive correlation with clinical features, while the blue module represented a negative correlation with clinical features (**Figure 5C**). Besides, AGs in the module of brown, turquoise, gray, and blue were significantly associated with clinical characteristics of BC. Interestingly, after taking the intersection of the 10 AGs in our risk model with the above four modules, it was found that eight AGs among the 10 AGs were identified to be related to BC, which indirectly verified the correlation of our risk model with clinical characteristics of BC (**Figure 5D**).

### The Correlation of the Risk Score With the Clinical Characteristics of Breast Cancer

**Figure 6:**
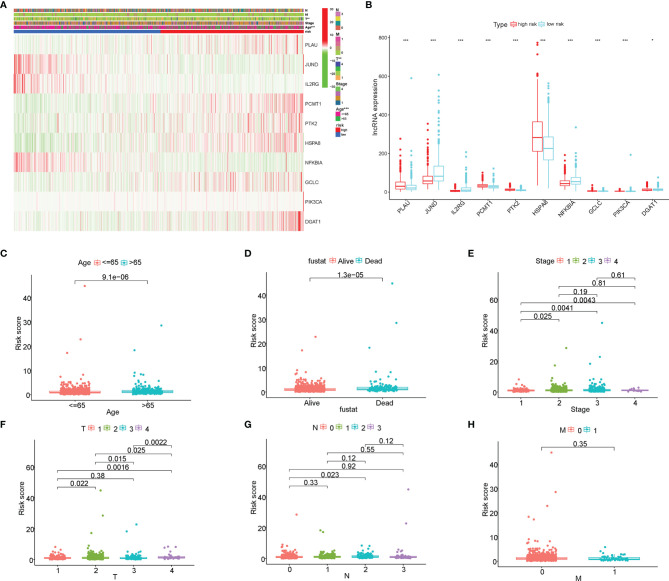
The correlation of the risk score with the clinical characteristics of breast cancer (BC). **(A)** The heatmap displayed the correlation between the 10 aging-related genes (AGs) and the clinical features of BC. **(B)** The expression of the 10 AGs is significantly different between high- and low-risk groups. **(C–H)** The scatter diagrams verified that the risk score had different distribution patterns in BC patients with different clinical characteristics, including age, survival status, clinical stage, and TNM staging. *P<0.05; **P<0.01; ***P<0.001.

The correlation of the 10 AGs with the clinical characteristics of BC was directly displayed *via* the heatmap (**Figure 6A**). Besides, those 10 AGs were different between between high- and low-risk groups (**Figure 6B**). Particularly, the high-risk group was enriched in the expression of *PLAU, PTK2, HSPA8*, and *GCLC*, while the low-risk group was enriched in the expression of *JUND, IL2RG, PCMT1, NFKBIA, PIK3CA*, and *DGAT1*. Meanwhile, we further analyzed the correlation between the risk score and different clinical factors by using the Wilcoxon signed-rank test. Interestingly, the scatter diagrams confirmed that the risk score exhibited differential distribution patterns in different clinical characteristics of BC patients, including age, survival status, clinical stage, and TNM staging (**Figures 6C**–**H**). In light of the molecular heterogeneity of BC, we discussed the independent prognostic value of risk score among the five molecular subtypes of BC. The results illustrated that in the lumA, lumB, Her2, basal-like, and normal subtypes, low-risk patients screened by risk score still had higher OS rates and longer OS durations, and both had P values <0.05 (**Figure S3**). The above results suggested that our risk score is closely related to the clinical features of BC and can be used as an effective auxiliary tool to predict the BC prognosis.

### The Gene Set Enrichment Analysis and Principal Component Analysis Between the High- and Low-Risk Groups

**Figure 7:**
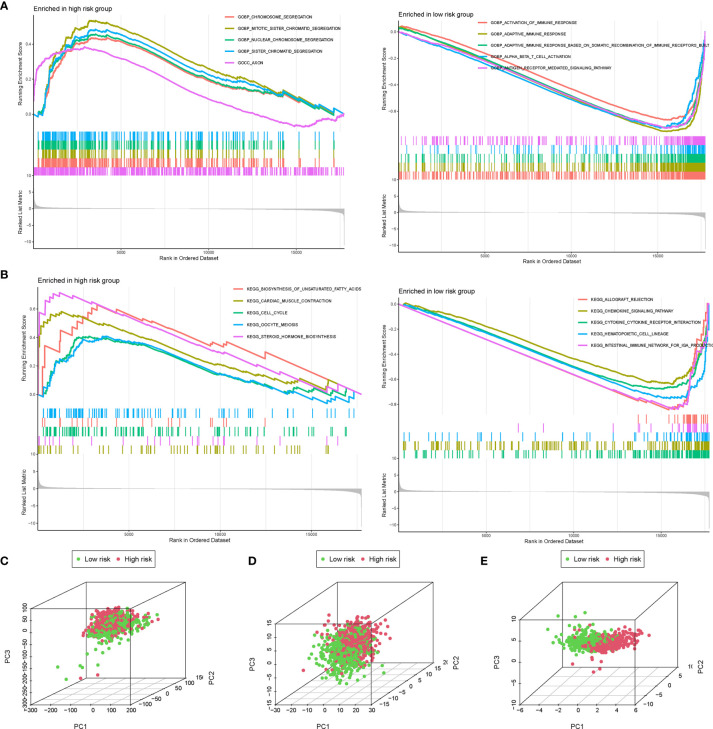
The gene set enrichment analysis (GSEA) and principal component analysis (PCA) between the high- and low-risk groups. The GSEA was performed to identify and visualize the differential Gene Ontology (GO) **(A)** and Kyoto Encyclopedia of Genes and Genomes (KEGG) **(B)** enrichment analysis between the high- and low-risk subgroups. PCA for the visualization of all genes **(C)**, all aging-related genes (AGs) **(D)**, and risk signature genes **(E)** to distinguish high- and low-risk subgroups in The Cancer Genome Atlas (TCGA) dataset.

The GSEA was further performed to identify the differential pathways enriched in GO and KEGG between the high- and low-risk subgroups. The GO analysis showed that the high-risk group was enriched in biological processes of genetic alteration, such as chromosome segregation, mitotic sister chromatid segregation, nuclear chromosome segregation, and sister chromatid segregation (**Figure 7A**). Besides, the KEGG analysis revealed that several processes, which were associated with tumor initiation and progression, such as biosynthesis of unsaturated fatty acids, cardiac muscle contraction, steroid hormone biosynthesis, and cell cycle, mainly occurred in the high-risk group (**Figure 7B**). Interestingly, both the GO and KEGG analyses found that the low-risk group was mainly enriched in immune-related biological processes and pathways. These findings proposed the distinction between the two risk subgroups, which was concentrated on genetic alteration, tumor progression, and immune response. Additionally, the PCA showed that compared to all genes (**Figure 7C**) and all AGs (**Figure 7D**), the risk model based on the 10 AGs clearly divided BC patients into two risk subgroups (**Figure 7E**).

### Genetic Alteration of Aging-Related Genes

**Figure 8:**
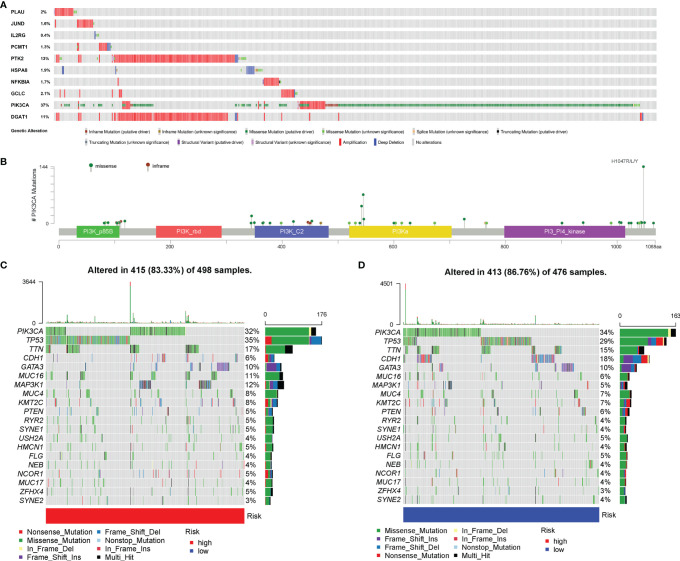
Genetic alteration of aging-related genes (AGs). **(A)** The analysis about the mutation status of the 10 AGs in our risk model. **(B)** The most frequent genetic alteration of *PIK3CA* was missense mutation and amplification. **(C)** The mutation frequency and classification of the first 20 gene mutations in the high-risk group. **(D)** The mutation frequency and classification of the first 20 gene mutations in the low-risk group.

After summarizing the incidence of CNVs and somatic mutations of all genes in BC, it was found that missense mutation was the most common variant classification, while the most common variant type was single-nucleotide polymorphisms (SNPs), and the top SNV class was C > T (**Figure S4**). The results also indicated that *TP53* and *PIK3CA* were the genes with the highest mutation frequency of 34% and 33%, respectively. Besides, the analysis of the mutation status of the 10 AGs in our risk model revealed that among those 10 AGs, *PIK3CA* had the most common genetic alterations (37%), and the most frequent genetic alteration was missense mutation and amplification **(** **Figure 8A**). Of the *PIK3CA* mutations, 357 (96.0%) were predicted to be missense mutations and 15 (4.0%) inframe mutations. In addition, the majority of *PIK3CA* mutations (216/724, 29.8%) were localized in the PI3-PI4 kinase (**Figure 8B**). Meanwhile, we further explored the mutation frequency and classification of all genes between the high- and low-risk groups of BC patients. It found that 415 of 498 (83.33%) BC samples in the high-risk group and 413 of 476 (86.76%) BC samples in the low-risk group displayed genetic mutations, and missense mutation was the most common variant classification (**Figures 8C, D**). Moreover, in the high-risk group, *PIK3CA* had high genetic alterations (32%), which was just junior to the genetic alterations of *TP53* (35%) (**Figure 8C**). In the low-risk group, *PIK3CA* had the most genetic alterations (34%) (**Figure 8D**).

### Analysis of Tumor Microenvironment and Tumor Immunity

**Figure 9:**
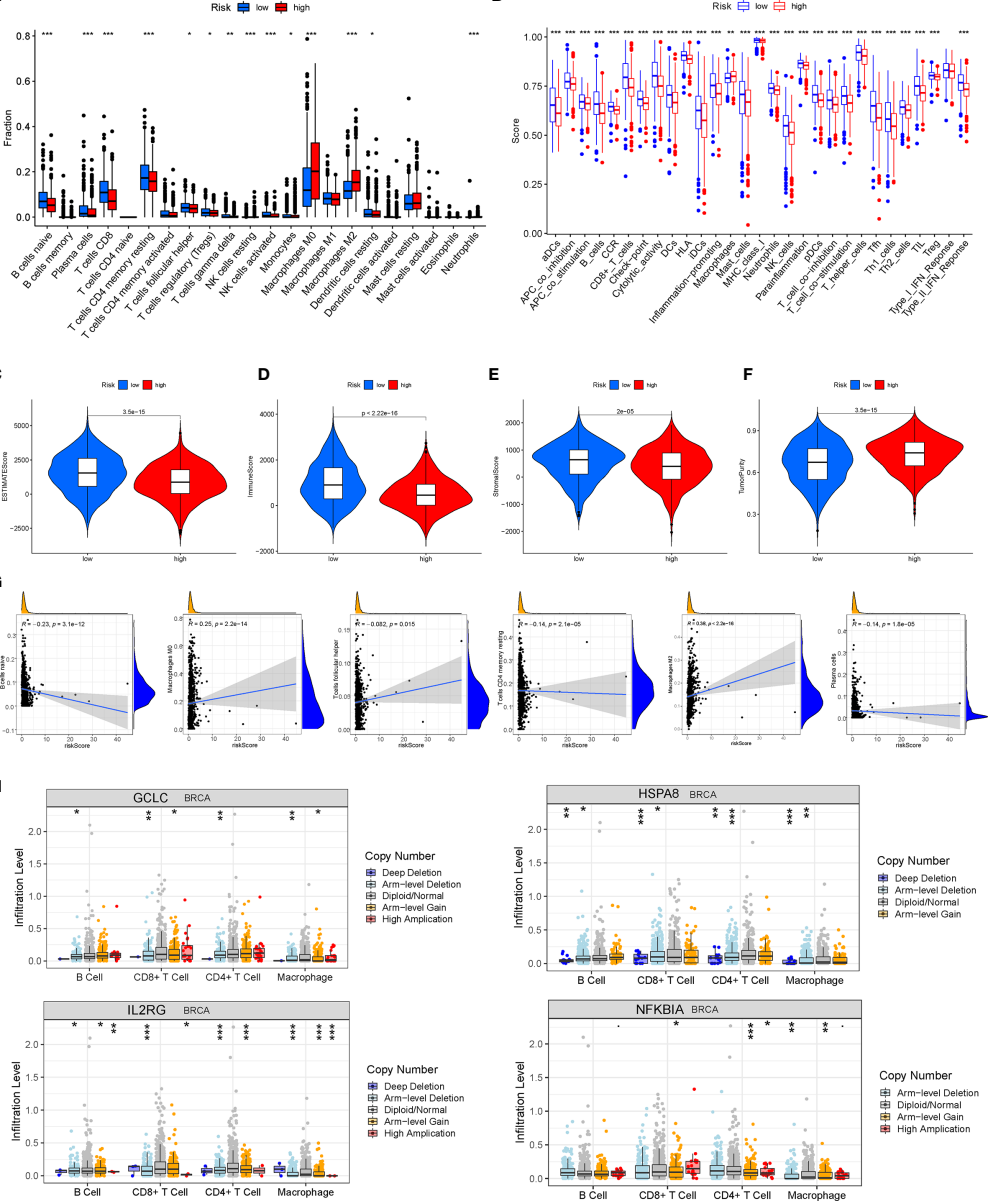
Analysis of tumor microenvironment (TME) and tumor immunity. The different distributions of the immune cell infiltration **(A)** and the immune function **(B)** between the high- and low-risk groups. **(C–F)** The Immune score, the Stromal score, the tumor purity, and the ESTIMATE score were applied to quantify the different immune statuses between the high- and low-risk groups. **(G)** Pearson’s correlation between six kinds of immune cells and the risk score. **(H)** The relationship between copy number variations (CNVs) of four aging-related genes (AGs) (*GCLC, HSPA8, IL2RG*, and *NFKBIA*) and the level of infiltration of B cells, T cells, and macrophages in breast cancer (BC). *P < 0.05; **P < 0.01; ***P < 0.001.

The ssGSEA was performed to quantify the enrichment scores of 22 immune cells and 13 immune cell-related functions between the two risk groups. Intriguingly, the enrichment of the immune cell infiltration between the high- and low-risk groups exhibited significantly different dimensionalities (**Figure 9A**). Moreover, the scores of some immune functions, such as antigen-presenting cell (APC) co-inhibition, chemokine receptor (CCR), cytolytic activity, checkpoint, and inflammation-promoting, were significantly enriched in the low-risk group (**Figure 9B**). Then, the violin plots were drawn to visualize the Immune score, the Stromal score, the tumor purity, and the ESTIMATE score (Stromal score combined with Immune score), which were used to quantify the different immune statuses between the high- and low- risk groups. The high-risk group had higher tumor purity, while the low-risk group had higher ESTIMATE scores, Immune scores, and Stromal scores, which demonstrated that the BC patients in the high-risk group had higher tumor purity and lower immune infiltration (**Figures 9C–F**). In addition, Pearson correlation analysis elucidated that six immune cells were significantly correlated with the risk score (P < 0.05) (**Figure 9G**). We further analyzed the correlation of immune cell infiltration with OS of BC patients and found that the infiltration of B-cell memory, T-cell follicular helper, macrophage M0, and macrophage M2 was associated with shorter survival time of BC patients (**Figure S5**). Moreover, the somatic copy number alterations (SCNA) module was used to explore the relationship between CNVs of the 10 AGs and the level of infiltration of B cells, T cells, and macrophages in BC. Intriguingly, the CNVs of *GCLC, HSPA8, IL2RG*, and *NFKBIA* were associated with the extent of immune infiltration in BC immune cells (**Figure 9H**), indicating that the above AGs might be targeted for immunotherapy.

### Analysis of Immunotherapy Effect, Methylation Patterns, and Drug Sensitivity

**Figure 10:**
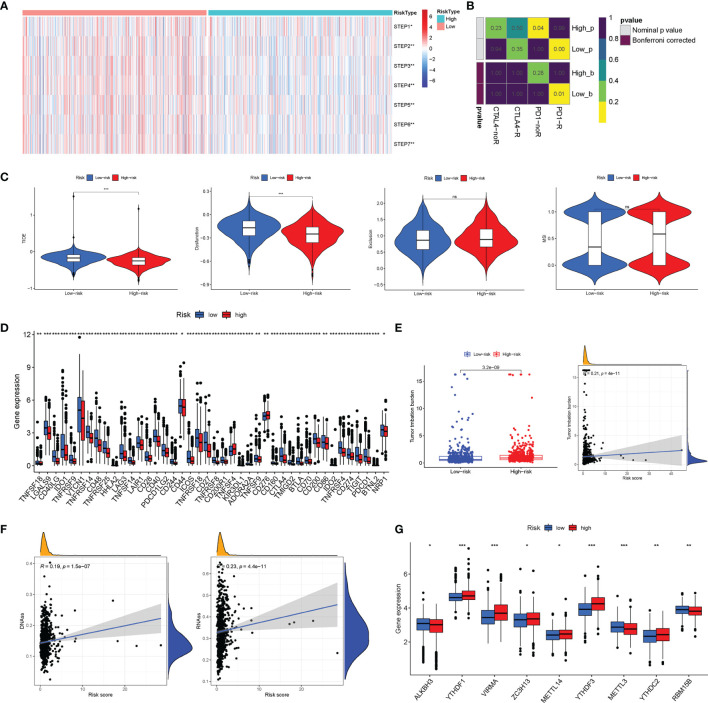
Analysis of immunotherapy effect and methylation patterns. **(A)** The possibility of anti-PD1 and anti-cytotoxic T lymphocyte-associated protein 4 (CTLA4) response to immunotherapy was evaluated between the high- and low-risk groups through the SubMAP algorithm. **(B)** The single-sample gene set enrichment analysis (ssGSEA) algorithm was performed to calculate the scores of the seven steps of the tumor-immune cycle. **(C)** The tumor immune dysfunction and exclusion (TIDE) algorithm was used to predict the response of The Cancer Genome Atlas (TCGA)-breast cancer (BC) patients to immunotherapy. **(D)** The distribution of immune checkpoints was significantly different between the high- and low-risk groups. **(E)** The BC patients in the high-risk group had higher tumor mutation burden (TMB) than that of BC patients in the low-risk group. **(F)** Pearson’s correlation between the RNA stemness score (RNAss) and DNA stemness score (DNAss) and the risk score. **(G)** The different expressions of m6A-related genes between the high- and low-risk groups. *P < 0.05; **P < 0.01; ***P < 0.001; ns (non significance).

Firstly, we calculated the scores of the seven steps of the tumor-immune cycle, and the results revealed that the low-risk group had the highest scores on the seven steps compared with those of the high-risk group (all P < 0.05) (**Figure 10A**). Subsequently, the SubMAP algorithm was applied to predict the possibility of response to anti-programmed death-1 (PD-1) and anti-cytotoxic T lymphocyte-associated protein 4 (CTLA4) response immunotherapy was evaluated between the high- and low-risk groups. The results revealed that the low-risk group may respond better to treatment (Bonferroni-corrected P = 0.01) (**Figure 10B**). Then, TIDE was further used to assess the potential immunotherapy effect in the two risk subgroups. The higher TIDE prediction score represented a higher possibility of immune escape, which suggested that the treatment effect of patients who received ICI was worse. In our study, the low-risk group had a higher TIDE score, proposing that BC patients in the high-risk group could benefit more from ICI therapy (**Figure 10C**). Besides, the low-risk group got a higher T-cell dysfunction score. However, there was no significant difference in T-cell exclusion score and microsatellite instability (MSI) between the two risk groups. Notably, the distribution of immune checkpoint-related molecule expression was significantly different between the high- and low-risk groups, which provided the potential targets of immunotherapy in BC patients with different risk scores (**Figure 10D**). Besides, BC patients in the high-risk group had higher TMB than that in patients in the low-risk group, supporting that BC patients in the high-risk groups were related to good outcomes of ICI treatments (**Figure 10E**). Moreover, the risk score was significantly positively correlated with TMB. That is, the efficacy of ICI treatment was enhanced in BC patients as the risk score increased. Intriguingly, the risk score was positively correlated with the RNA stemness score (RNAss) and DNA stemness score (DNAss), uncovering that the mRNA expression and DNA methylation patterns in BC patients altered as the risk score changed (**Figure 10F**). Besides, m6A-related genes were expressed significantly differentially between the two risk subgroups, suggesting that BC patients’ different risk scores were accompanied by the changes of m6A modification patterns (**Figure 10G**). Furthermore, the correlation analysis was performed between 263 chemotherapeutic drugs and the expression of the 10 AGs in our risk model. As shown in **Figure S6**, there existed a significant association of our risk AGs with the most common chemotherapeutic drugs, which provided potential guidance for chemotherapy in drug choice based on AGs.

### Validation of the Predictive Ability of the Risk Model in a Clinical External Cohort

**Figure 11:**
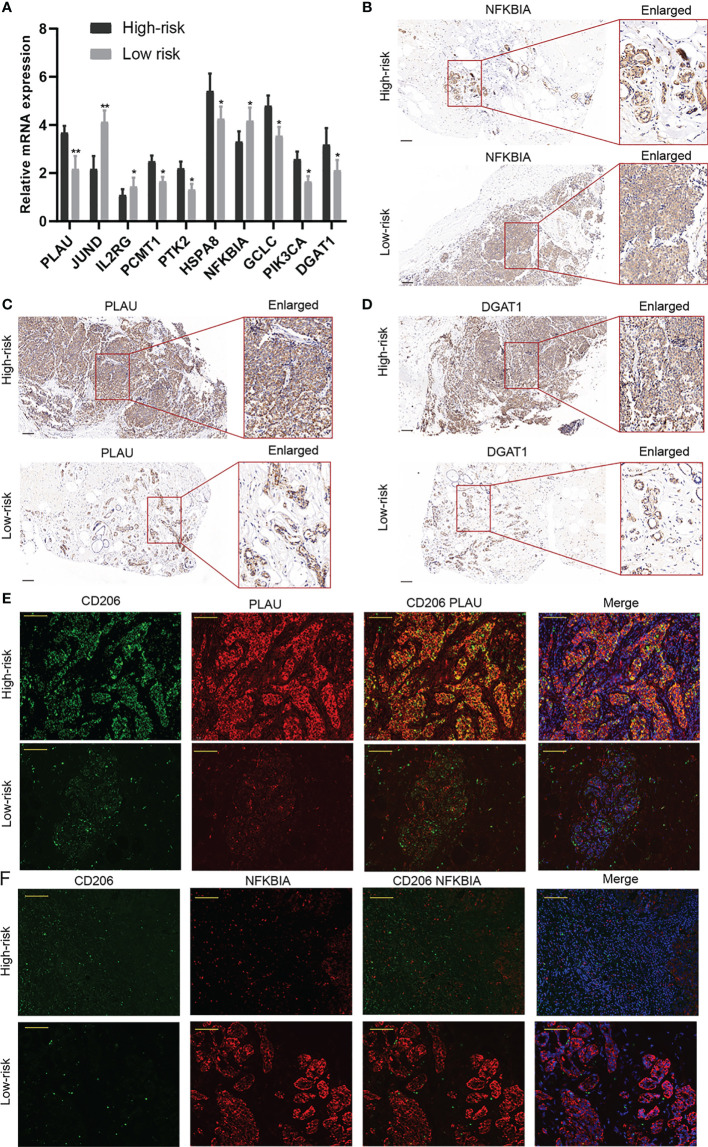
Validation of the predictive ability of the risk model in a clinical external cohort. **(A)** The relative expressions of the 10 aging-related genes (AGs) in our risk model were assessed in a clinical external cohort through qRT-PCR. **(B–D)** The results of immunohistochemistry (IHC) revealed that the expressions of PLAU and DGAT1 were increased but NFKBIA expression was decreased in the high-risk group. **(E, F)** The immunofluorescence (IF) assay demonstrated that M2 macrophages were more abundant in high-risk patients. *P < 0.05; **P < 0.01.

A clinical cohort that includes 20 BC patients with different clinical stages was constructed to verify the predictive ability of the risk score. Firstly, the relative expressions of the 10 AGs in our risk model were remarkably different between BC in different stages through qRT-PCR (**Figure 11A**). Then, PLAU, NFKBIA, and DGAT1 were detected between high- and low-risk groups, and CD206 was applied to indicate the relative abundance of M2 macrophages. The results of IHC further confirmed that the expressions of PLAU and DGAT1 were increased but NFKBIA expression was decreased in the high-risk group in comparison to those of the low-risk group patients (**Figures 11B**–**D**). The IF assay further demonstrated that M2 macrophages were more abundant in the high-risk patients, and CD206 and PLAU were more co-expressed in high-risk patients (**Figures 11E, F**).

### DGAT1 Promoting the Proliferation and Migration of Breast Cancer Cells

**Figure 12:**
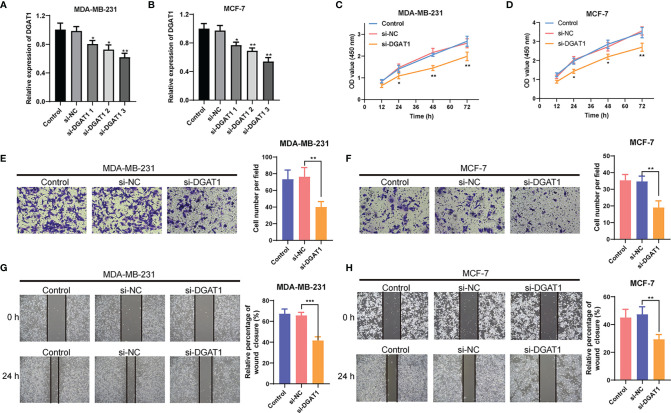
The effect of DGAT1 on proliferation and migration of breast cancer (BC) cells. **(A, B)** The results of qRT-PCR showed that siRNAs could successfully knock down the expression of DGAT1 in MCF-7 and MDA-MB-231. **(C, D)** The depletion of DGAT1 observably suppressed the proliferation ability in both MCF-7 and MDA-MB-231 compared with the si-NC group. **(E–H)** The knockdown of DGAT1 could significantly decrease the migration of MCF-7 and MDA-MB-231 BC cells. *P < 0.05; **P < 0.01; ***P < 0.001.

To further explore the role of DGAT1 in BC cells, we examined whether the knockdown of *DGAT1* gene could affect the proliferation and migration of MCF-7 and MDA-MB-231 BC cells. As shown in **Figures 12A, B**, siRNAs could successfully knock down the expression of *DGAT1* in MCF-7 and MDA-MB-231. The CCK-8 analysis revealed that the depletion of DGAT1 observably suppressed the proliferation ability in both MCF-7 and MDA-MB-231 compared with the si-NC group (**Figures 12C, D**). Meanwhile, the transwell migration assay and wound healing assay were performed to investigate the impact of DGAT1 on BC cell migration. The results demonstrated that knockdown of *DGAT1* could significantly decrease the migration of MCF-7 and MDA-MB-231 BC cells (**Figures 12E–H**).

## Discussion

The intratumor heterogeneity in BC is a well-known concept that has been confirmed in different therapeutic regimen sensibilities and clinical outcomes among different BC subtypes. Many studies on various cancers have shown the importance of aging in tumorigenesis and its potential application in cancer risk prediction (29). Aging has been acknowledged as a dominant risk factor for malignant processes in BC. For prognostic classification, it would be of greater predictive ability to build a risk model based on a specific AG signature than a single gene in BC. The main purpose of this study is to establish a predictive risk model based on AGs for predicting survival outcomes in patients with BC. Specifically, we performed a systematic investigation of the associations between AGs and BC prognosis and constructed a prognosis risk model with 10 AGs (including *PLAU, JUND, IL2RG, PCMT1, PTK2, HSPA8, NFKBIA, GCLC, PIK3CA*, and *DGAT1*). Moreover, our analysis further confirmed that the nomogram possessed a robust performance signature for predicting prognosis compared to clinical characteristics of BC patients, including age, survival status, clinical stage, and TNM stage.

As aging is emerging as a crucial risk factor for cancer, there have been several reports in constructing prognostic assessment tools. For example, Xu and Chen (30) utilized a six AG-based pattern (*APOC3, EPOR, H2AFX, MXD1, PLCG2*, and *YWHAZ*) to structure a risk model that could efficiently stratify the existing cases in high- and low-risk groups in terms of OS in lung adenocarcinoma (LUAD). In addition, Yang et al. (31) constructed a risk model based on seven AGs (*APP, CDKN2A, EGFR, HSPD1, IL2RG, PLAU*, and *VEGFA*) for head and neck squamous cell carcinoma (HNSCC) prognostic evaluation. They found that the high-risk score was significantly related to immunosuppression and pro-inflammatory factors [interleukin (IL)-1α, IL-1β, IL-6, and IL-8]. Similarly, Chen et al. (32) also identified seven AGs (*POLA1, CDK1, SOCS2, HDAC1, MAPT, RAE1*, and *EEF1E1*) to establish a risk model in evaluating the hepatocellular carcinoma (HCC) prognosis. The high-risk scores indicated a lower performance of tumor differentiation, higher stage, downregulation of metabolism, and tumor immunity and worse prognosis in HCC patients. Luo et al. (33) reported a model constructed by a profile of survival-associated AGs (*LEP*, *TERT*, *PON1*, and *SSTR3*) in TCGA dataset and Chinese Glioma Genome Atlas (CGGA), confirming that the risk score was an independent prognostic factor and was closely tied to tumor immune microenvironment in glioma.

In our study, the 10 AG-based model could effectively divide the included patients into high- and low-risk groups, indicating the shorter DFS, PFI, and OS in the high-risk group. Risk scores showed different distribution patterns among different clinical characteristics of BC patients. These AGs were confirmed to participate in the process of BC oncogenesis and progression. More importantly, these AGs were closely associated with the mutation of *TP53* and *PIK3CA*. There are higher tumor purity and lower immune infiltration in the high-risk group. Our study is further proof of AG-based model reliability, which was consistent with the above reports. The difference between previous studies was that here we adopted a more comprehensive quantity of the 10 AGs in the setting of BC, which also acquired good predictive results and evaluation of immune infiltration and immunotherapy. The external clinical sample validation indicated that AGs were differentially expressed in patients in the high- and low-risk groups, and tumor-associated macrophage marker CD206 was elevated in high-risk BC tissues with more co-localization of AGs. DGAT1 is a transmembrane protein found in the endoplasmic reticulum of several cell types. It has been proven that DGAT1 could promote tumor progression in several cancer types, such as ovarian and prostate cancer, but the role of DGAT1 in BC is rarely studied (34, 35). Therefore, we verified the effect of DGAT1 in BC cells, and the results confirmed the promoting effects of DGAT1 in BC cell proliferation and migration, which were consistent with the results in other cancer types. Consequently, these tumor prediction models based on AGs are indeed precisely for predicting BC prognosis and making a treatment decision.

In regard to the immunobiology of BC, emerging biomarkers such as TMB and TIL infiltration are worth exploring in depth. Despite the heterogeneity of immune cell type components, the average TILs in the pathological staining section stanchly reflected the TILs in the whole tumor, which were markedly associated with outcome in triple-negative breast cancer (TNBC) patients, as well as possessing prognostic significance for tumor relapse (36). Karn et al. (37) showed that the lymphocyte-rich TNBCs with good prognosis presented a significantly decreased expression of mutation and neoantigen counts in comparison to the lymphocyte-poor TNBCs with poor prognosis. Interestingly, in our study, it found that resting memory CD4^+^ T cells, naive B cells, and plasma cells were associated with poor prognosis in BC, which was in keeping with the previous investigations. Therefore, impaired antitumor immune function may account for the poor prognosis in high-risk patients. The majority of AGs included in our signature are closely related to tumor initiation, proliferation, and metastasis. Besides, there was a strong correlation between prediction score and immunotherapy effect, methylation patterns, and drug sensitivity, posing the prospect of these AGs in BC individualized treatments. Therefore, the AG-based risk model benefits the prognosis stratification and ICI therapy in favor of BC management.

Meanwhile, there are still some issues to be addressed in our study. First, although the predictive capability of this risk model has been systematically evaluated in the databases with multiple analytical methods, it is still a retrospective study. It would be of value to continue more independent prospective studies in a large cohort, which could further validate the predictive capability in real-world data. Secondly, the AG-based risk model was closely related to the immune state of BC. Further studies are needed to assess the underlying mechanisms associated with inflammatory infiltration of the identified AGs in BC.

## Conclusion

In the current study, we successfully established a risk model for BC prognosis, which was constituted by a signature of 10 AGs and possessed the predictive ability in BC diagnosis and immunotherapy status. This study offered a novel insight into the reliable integrated model with multiple genes in BC prognostic prediction.

## Data Availability Statement

The datasets presented in this study can be found in online repositories. The names of the repository/repositories and accession number(s) can be found in the article/**Supplementary Material**.

## Author Contributions

YW, NZ, and QZ conceived the project. WL mainly analyzed the data and CZ mainly conducted the experiments. Both WL and CZ wrote and revised the article. YT performed the literature investigation and revised the article, including figures and tables. YW provided support for the construction of the outline. WH and HY assisted in revising the article. All authors have reviewed the article and all approved the final version.

## Funding

This work was supported by the China Guanghua Science and Technology Foundation (grant number: 2019JZXM001) and Wuhan Science and Technology Bureau (grant number: 2020020601012241).

## Conflict of Interest

The authors declare that the research was conducted in the absence of any commercial or financial relationships that could be construed as a potential conflict of interest.

## Publisher’s Note

All claims expressed in this article are solely those of the authors and do not necessarily represent those of their affiliated organizations, or those of the publisher, the editors and the reviewers. Any product that may be evaluated in this article, or claim that may be made by its manufacturer, is not guaranteed or endorsed by the publisher.

## Supplementary Material

Supplementary Figure 1 The Kaplan-Meier analysis revealed that among those 10 AGs, PLAU, PCMT1, PIK3CA, PTK2, HSPA8, GCLC, and DGAT1 were correlated with poor OS of BC patients.

Supplementary Figure 2 The ROC analysis and the C-index were used to evaluate the reliability and superiority of our risk score signature against other published signatures.

Supplementary Figure 3 The Kaplan-Meier analysis indicated that in the lumA, lumB, Her2, basal-like, and normal subtypes, low-risk patients screened by risk score had higher OS rates and longer OS durations (P < 0.05).

Supplementary Figure 4 Visualization of the incidence of CNVs and somatic mutations of all genes in BC.

Supplementary Figure 5 The Kaplan-Meier analysis revealed that the infiltration of B cells memory, T cells follicular helper, B cells naïve, Plasma cells, Macrophages M0, Macrophages M2, and T cells CD4 memory resting were associated with survival time of BC patients.

Supplementary Figure 6 Scatter plot of the relationship between AGs expression and drug sensitivity (the top 16 correlations with the lowest P values were displayed).

## Glossary

BC: breast cancer
AGs: aging-related genes
TCGA: The Cancer Genome Atlas
LASSO: least absolute shrinkage and selection operator
GO: Gene Ontology
KEGG: Kyoto Encyclopedia of Genes and Genomes
OS: overall survival
DFS: disease-free survival
PFI: progression-free interval
WGCNA: weighed gene co-expression network analysis
GSEA: gene set enrichment analysis
ssGSEA: single-sample gene set enrichment analysis
PCA: principal component analysis
CIBERSORT: Cell-type Identification by Estimating Relative Subsets of RNA Transcripts
TME: tumor microenvironment
TIDE: tumor immune dysfunction and exclusion
ICI: immune checkpoint inhibitor
TMB: tumor mutation burden
cBioPortal: cBio Cancer Genomics Portal database
CNVs: copy number variations
siRNAs: small interference RNAs
CCK-8: Cell Counting Kit-8
OD: optical density
PBS: phosphate-buffered saline
IHC: immunohistochemistry
IF: immunofluorescence
HRP: horseradish peroxidase
ROC: receiver operating characteristic
AUC: area under the curve
SD: standard deviation
DEAGs: differentially expressed aging-related genes
CTLA4: cytotoxic T lymphocyte-associated protein 4
MSI: microsatellite instability
RNAss: RNA stemness score
DNAss: DNA stemness score
CGGA: Chinese Glioma Genome Atlas
