# Supplementary figures and images for: Identification of an Aging-Related Gene Signature in Predicting Prognosis and Indicating Tumor Immune Microenvironment in Breast Cancer
Source: Front Oncol. 2021 Dec 16;11:796555. doi: 10.3389/fonc.2021.796555 (PMC8716799; doi:10.3389/fonc.2021.796555)

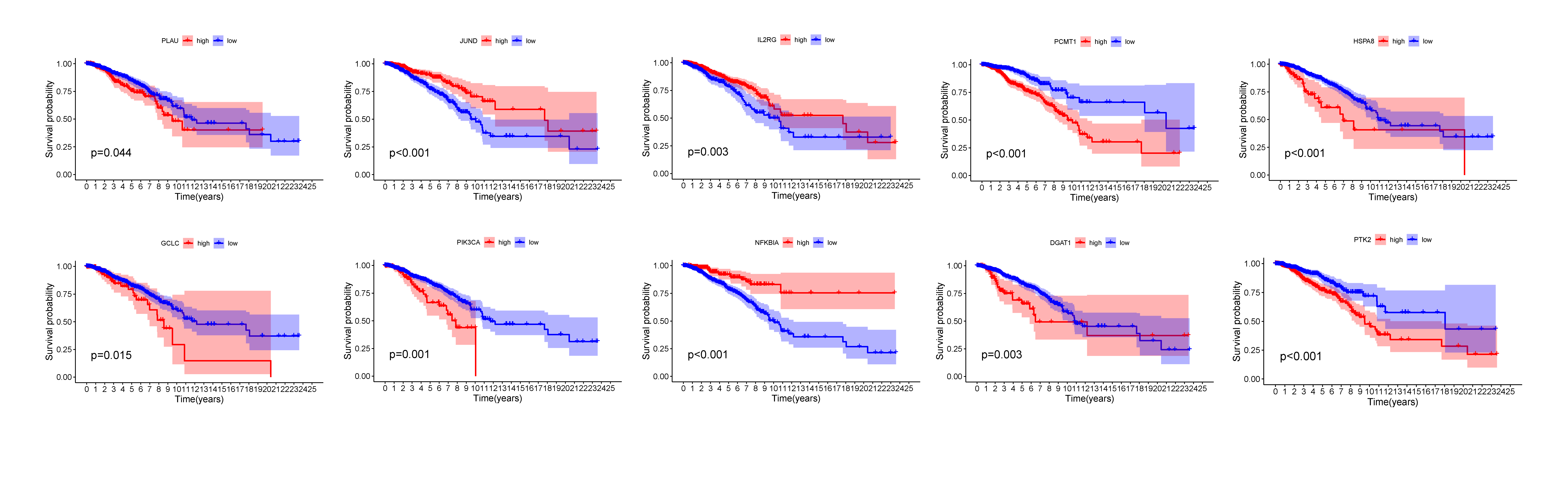

Supplement: Supplementary Figure 1 — The Kaplan-Meier analysis revealed that among those 10 AGs, PLAU, PCMT1, PIK3CA, PTK2, HSPA8, GCLC, and DGAT1 were correlated with poor OS of BC patients. [file Image_1.tiff]

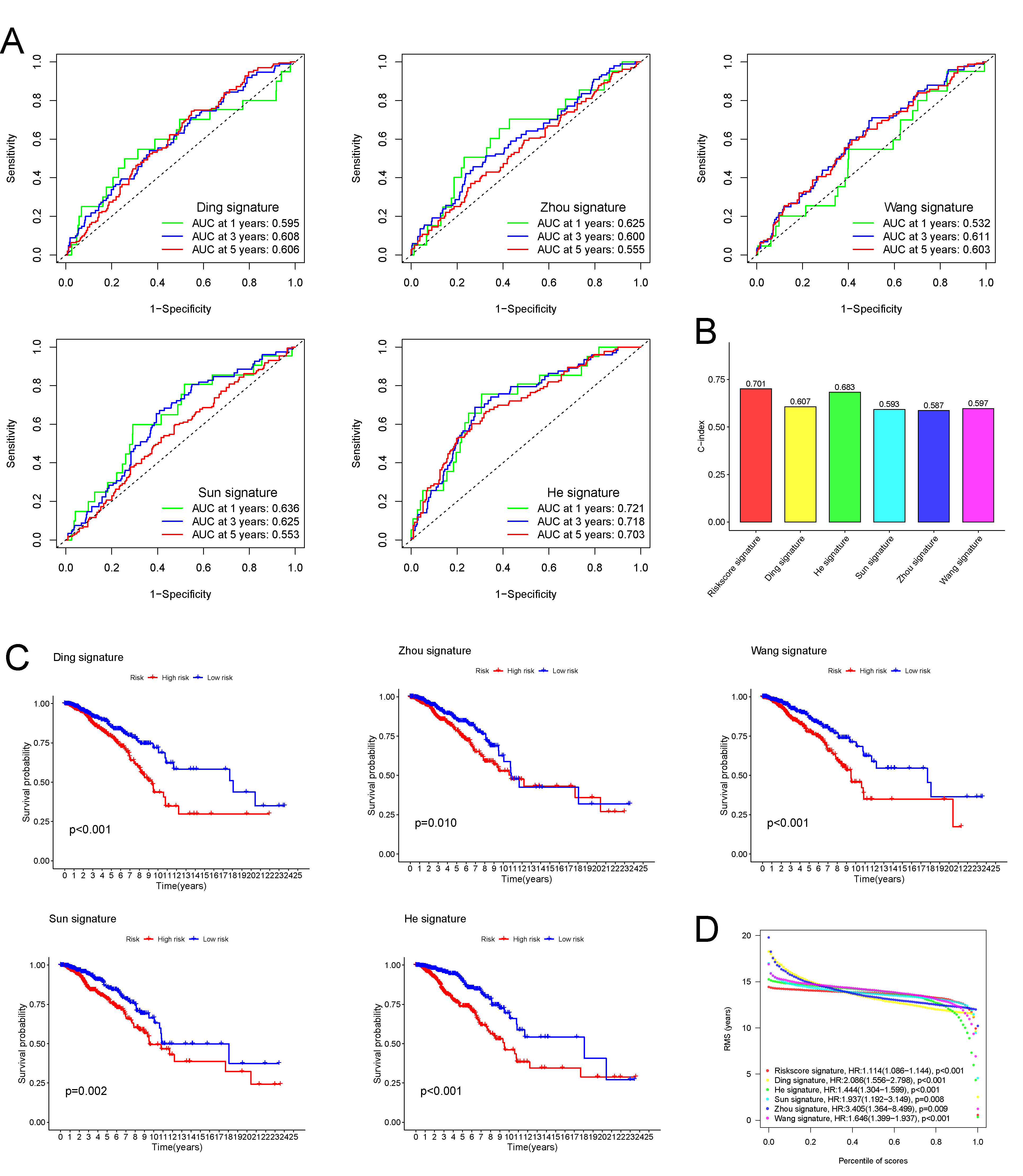

Supplement: Supplementary Figure 2 — The ROC analysis and the C-index were used to evaluate the reliability and superiority of our risk score signature against other published signatures. [file Image_2.tif]

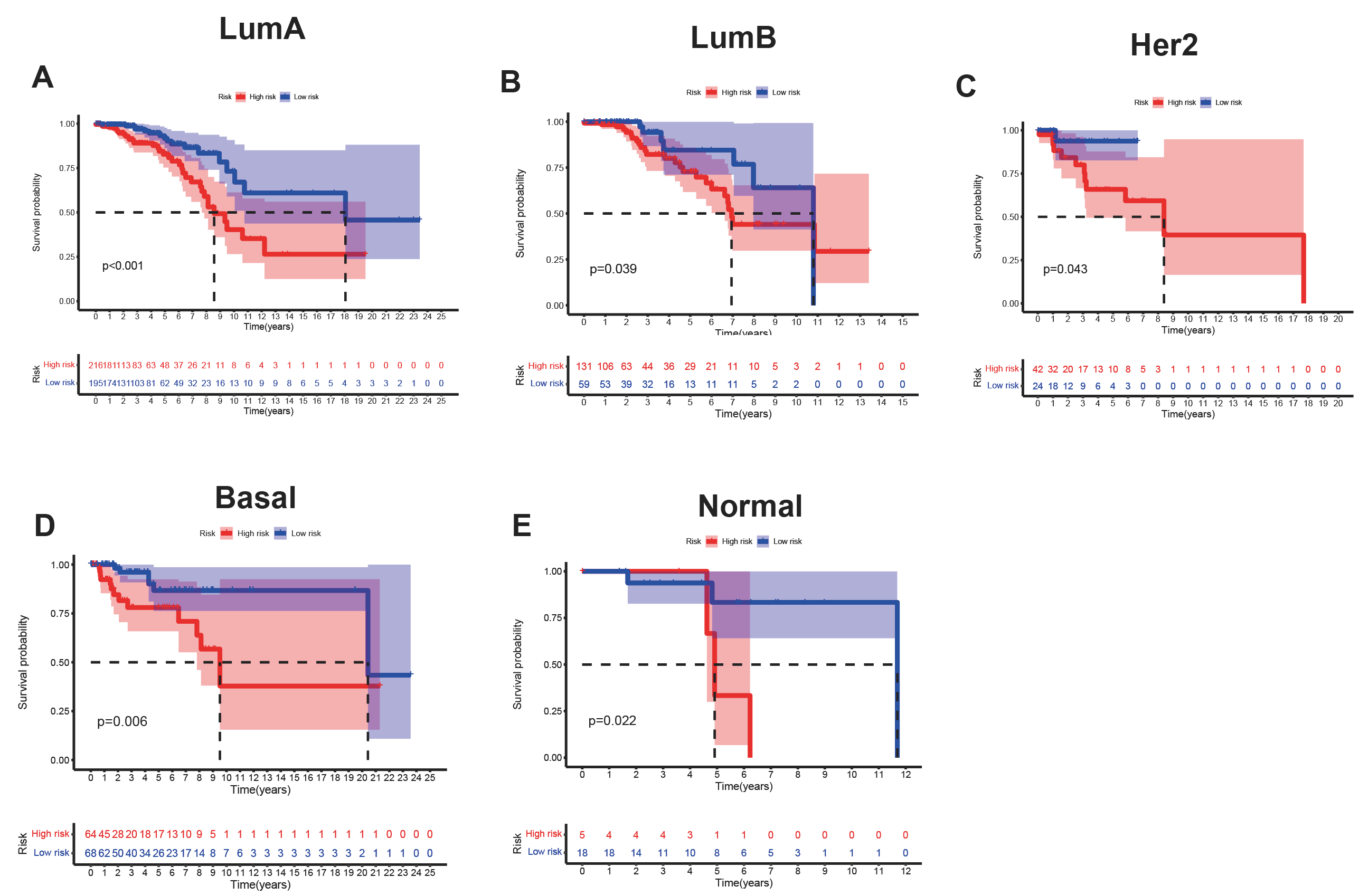

Supplement: Supplementary Figure 3 — The Kaplan-Meier analysis indicated that in the lumA, lumB, Her2, basal-like, and normal subtypes, low-risk patients screened by risk score had higher OS rates and longer OS durations (P < 0.05). [file Image_3.tif]

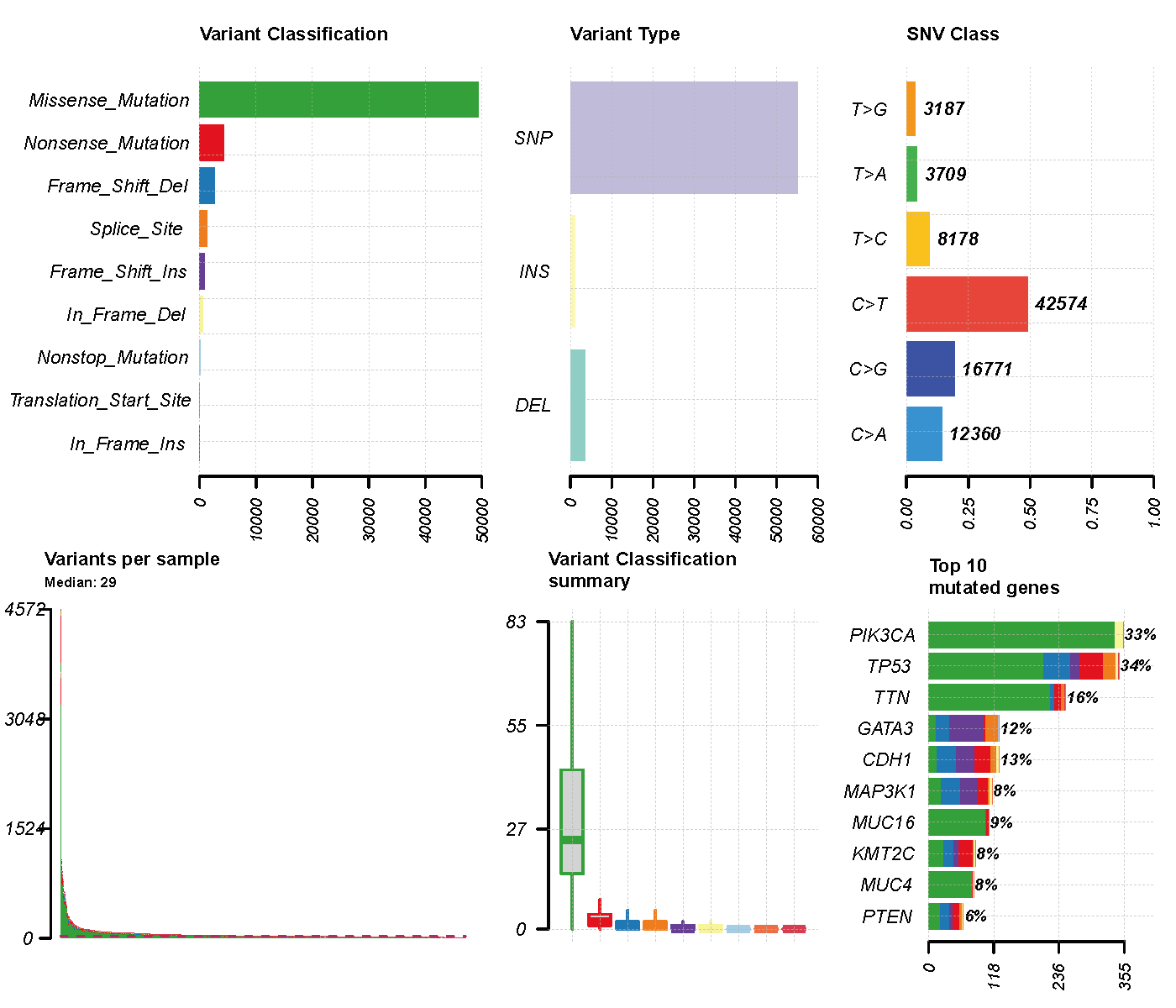

Supplement: Supplementary Figure 4 — Visualization of the incidence of CNVs and somatic mutations of all genes in BC. [file Image_4.tif]

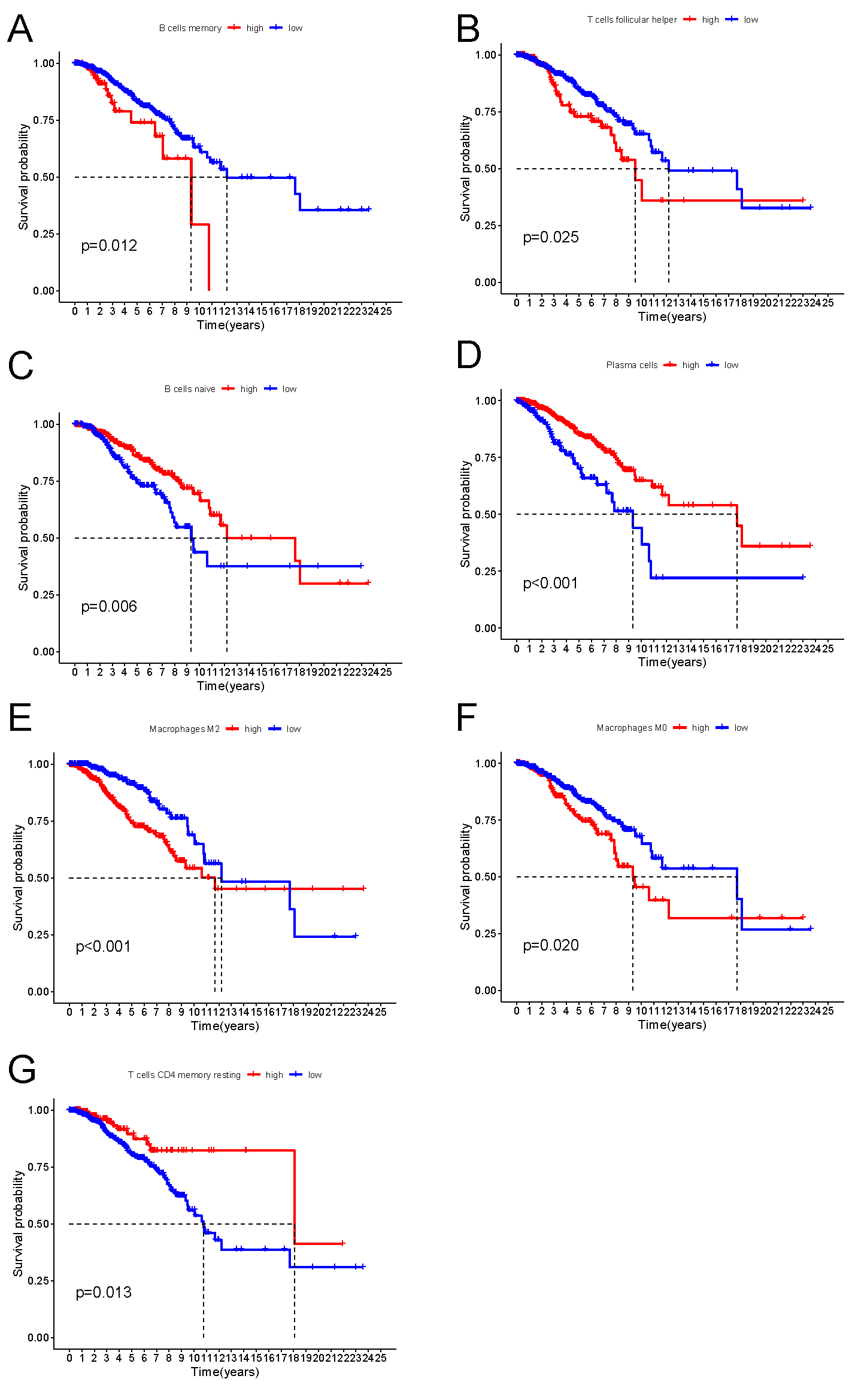

Supplement: Supplementary Figure 5 — The Kaplan-Meier analysis revealed that the infiltration of B cells memory, T cells follicular helper, B cells naïve, Plasma cells, Macrophages M0, Macrophages M2, and T cells CD4 memory resting were associated with survival time of BC patients. [file Image_5.tiff]

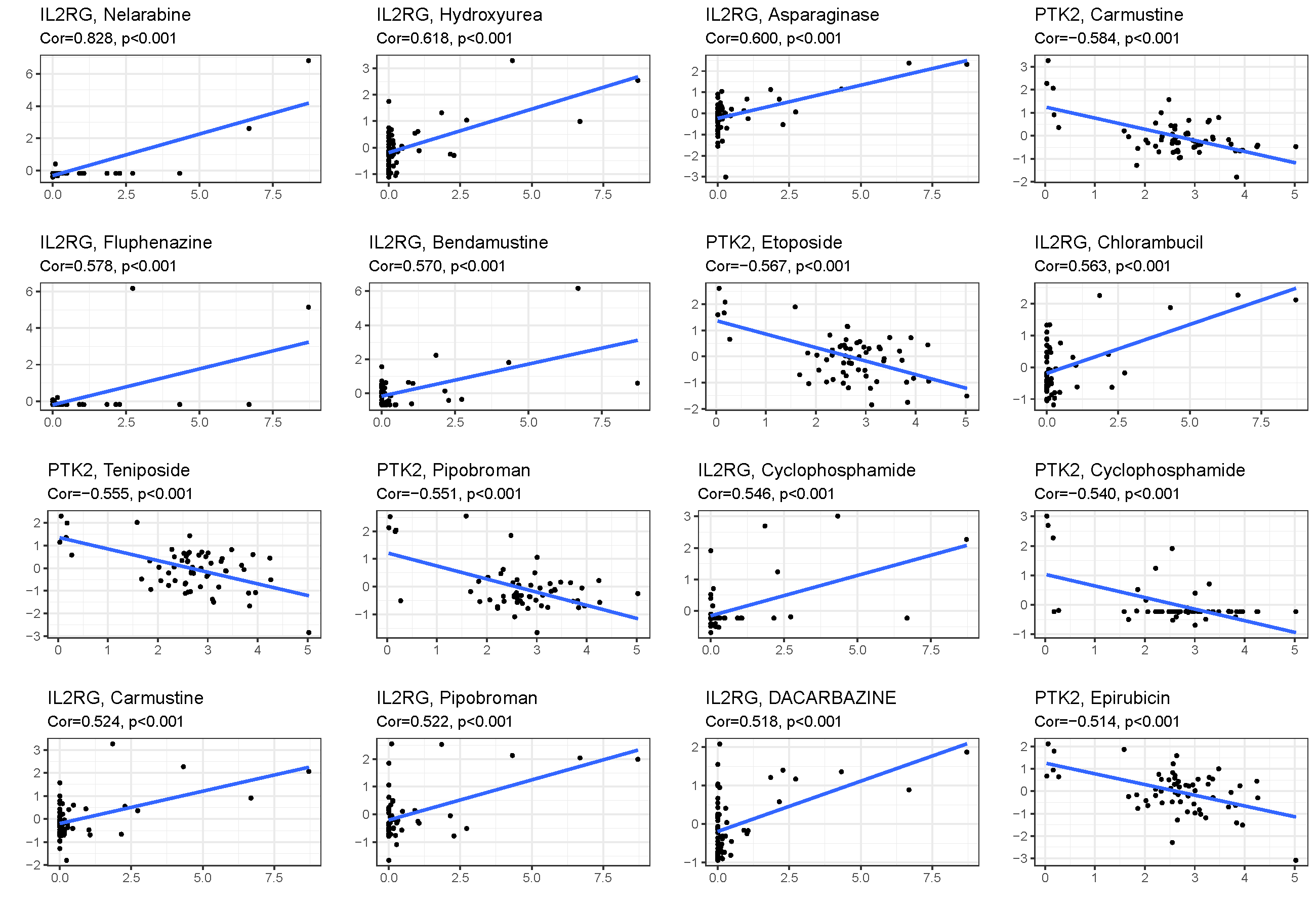

Supplement: Supplementary Figure 6 — Scatter plot of the relationship between AGs expression and drug sensitivity (the top 16 correlations with the lowest P values were displayed). [file Image_6.tiff]
