# Supplementary material for: Identification of an Aging-Related Gene Signature in Predicting Prognosis and Indicating Tumor Immune Microenvironment in Breast Cancer
Source: Front Oncol. 2021 Dec 16;11:796555. doi: 10.3389/fonc.2021.796555 (PMC8716799; doi:10.3389/fonc.2021.796555)
Supplement: Supplementary file 7 [file Table_1.docx]

| **Characteristic** | **external validation cohort data (n = 20)** |
| --- | --- |
| **Age, n (%)** |  |
| < 65 | 13 (65) |
| ≥ 65 | 7 (35) |
| **WHO-Stage, n (%)** |  |
| Ⅰ | 5 (25) |
| Ⅱ | 4 (20) |
| Ⅲ | 10 (50) |
| Ⅳ | 1 (5) |
| **AJCC-T stage, n (%)** |  |
| T1 | 5 (25) |
| T2 | 3 (15) |
| T3 | 7 (35) |
| T4 | 5 (25) |
| **AJCC-N stage, n (%)** |  |
| N0 | 4 (20) |
| N1 | 2 (10) |
| N2 | 9 (45) |
| N3 | 5 (25) |
| **AJCC-M stage, n (%)** |  |
| M0 | 19 (95) |
| M1 | 1 (5) |

**Supplementary Table 1. Clinical characteristics of BC patients in external validation cohort**
