# Supplementary material for: Identification of an Aging-Related Gene Signature in Predicting Prognosis and Indicating Tumor Immune Microenvironment in Breast Cancer
Source: Front Oncol. 2021 Dec 16;11:796555. doi: 10.3389/fonc.2021.796555 (PMC8716799; doi:10.3389/fonc.2021.796555)
Supplement: Supplementary file 9 [file Table_3.doc]

| **Supplementary Table 3. qPCR primer sequence** | | | |
| --- | --- | --- | --- |
| mRNA | Species | Forward | Reverse |
| PLAU | Human | GCTTGTCCAAGAGTGCATGGT | CAGGGCTGGTTCTCGATGG |
| JUND | Human | TCATCATCCAGTCCAACGGG | TTCTGCTTGTGTAAATCCTCCAG |
| IL2RG | Human | GTGCAGCCACTATCTATTCTCTG | GTGAAGTGTTAGGTTCTCTGGAG |
| PCMT1 | Human | CTGAAGGTGGTTCTGTACCTGC | GGAGATTGTGGATTAGCTCCGA |
| PTK2 | Human | GCTTACCTTGACCCCAACTTG | ACGTTCCATACCAGTACCCAG |
| HSPA8 | Human | ACCTACTCTTGTGTGGGTGTT | GACATAGCTTGGAGTGGTTCG |
| NFKBIA | Human | CTCCGAGACTTTCGAGGAAATAC | GCCATTGTAGTTGGTAGCCTTCA |
| GCLC | Human | GGAGGAAACCAAGCGCCAT | CTTGACGGCGTGGTAGATGT |
| PIK3CA | Human | CCACGACCATCATCAGGTGAA | CCTCACGGAGGCATTCTAAAGT |
| DGAT1 | Human | TATTGCGGCCAATGTCTTTGC | CACTGGAGTGATAGACTCAACCA |
| GAPDH | Human | GGAGCGAGATCCCTCCAAAAT | GGCTGTTGTCATACTTCTCATGG |
